# Impact of Social Media Influencers on Amplifying Positive Public Health Messages

**DOI:** 10.2196/73062

**Published:** 2025-03-21

**Authors:** Gerard Thomas Flaherty, Ryan Michael Mangan

**Affiliations:** 1 School of Medicine College of Medicine, Nursing and Health Sciences Ollscoil na Gaillimhe – University of Galway Galway Ireland

**Keywords:** social media, COVID-19, vaccination, personal brands, public health, wellness, global health, pandemic, Twitter, tweets, vaccine, longitudinal design, wellness influencers, hand annotation, antivaccination, infodemiology

To the Editor-in-Chief,

Social media platforms have become powerful tools for disseminating health information. The recent study published in this journal by O’Brien et al [1] was of great interest. The authors explored the role of social media influencers in sharing health content, assessing their impact on online followers [1]. While the study found that many social media influencers resisted public health campaigns, especially those regarding COVID-19 vaccine uptake, it also highlighted their capacity to promote vaccine uptake when they are aligned with public health messaging [1]. Drawing from our collective experience in this field, we wish to highlight the significant potential of social media influencers in spreading positive public health messages, provide examples of their influence, and propose strategies to maximize their effectiveness in public health campaigns.

Social media influencers play a critical role in shaping public opinion on a wide range of health topics, including vaccinations, cancer screening, lifestyles, and travel safety [2-4]. Social media influencers can effectively counter vaccine hesitancy caused by misinformation, leading to improvements in community attitudes toward influenza vaccines among ethnic minority populations, for example [3]. Additionally, social media use has been linked to improvements in health and food literacy, particularly among schoolchildren [2]. Furthermore, health professionals who create content themselves recognize the value of social media influencers in disseminating information, especially when these influencers work within the boundaries of accurate and responsible messaging [4].

The so-called accessibility influencers also play a role in raising awareness and advocating for inclusive digital experiences by transforming complex health information into more engaging and understandable content [5]. Their work demonstrates the potential for social media influencers to drive positive societal change. The field of accessibility has evolved from its initial focus on web accessibility to addressing diverse user needs across digital platforms. While influencers focus on raising awareness and advocating for accessibility, they do not have a formal role in the development of standards or policies. Influencers’ personal experiences and community engagement underscore the importance of collaboration, demonstrating how social media can be harnessed for the public good [5].

Health expert content creators provide valuable insights into the evolving role of social media influencers in health communication. While they acknowledge the risks of misleading health content, which could confuse audiences or make health information less applicable to a broader population [4], they have also suggested strategies to guide social media influencers more effectively. These include improving public health and social media literacy to help audiences critically evaluate online health content, providing training for content creators to enhance the accuracy of their messages, and strengthening legal or platform-based regulations to curb the spread of misinformation. They also advocate the verification of health professionals’ credentials and encourage them to take a more active role on social media [4].

There is a need for further research to fully understand the impact of social media influencers on health communication. The report from O’Brien et al [1] adds to the ongoing discussion about the potential role of digital influencers and emphasizes the need for a cooperative approach to ensure that their influence is used constructively for the benefit of public health.
